# Alzheimer’s amyloid β heterogeneous species differentially affect brain endothelial cell viability, blood‐brain barrier integrity, and angiogenesis

**DOI:** 10.1111/acel.13258

**Published:** 2020-11-06

**Authors:** Rebecca Parodi‐Rullán, Jorge Ghiso, Erwin Cabrera, Agueda Rostagno, Silvia Fossati

**Affiliations:** ^1^ Alzheimer's Center at Temple Lewis Katz School of Medicine Temple University Philadelphia PA USA; ^2^ Department of Pathology New York University School of Medicine New York NY USA; ^3^ Department of Psychiatry New York University School of Medicine New York NY USA; ^4^ Present address: Farmingdale State College State University of New York Farmingdale NY USA

**Keywords:** Alzheimer's disease, amyloid β, angiogenesis, blood‐brain barrier, cerebral amyloid angiopathy

## Abstract

Impaired clearance in the Alzheimer's Disease (AD) brain is key in the formation of Aβ parenchymal plaques and cerebrovascular deposits known as cerebral amyloid angiopathy (CAA), present in >80% of AD patients and ~50% of non‐AD elderly subjects. Aβ deposits are highly heterogeneous, containing multiple fragments mostly derived from catabolism of Aβ40/Aβ42, which exhibit dissimilar aggregation properties. Remarkably, the role of these physiologically relevant Aβ species in cerebrovascular injury and their impact in vascular pathology is unknown. We sought to understand how heterogeneous Aβ species affect cerebral endothelial health and assess whether their diverse effects are associated with the peptides aggregation propensities. We analyzed cerebral microvascular endothelial cell (CMEC) viability, blood‐brain barrier (BBB) permeability, and angiogenesis, all relevant aspects of brain microvascular dysfunction. We found that Aβ peptides and fragments exerted differential effects on cerebrovascular pathology. Peptides forming mostly oligomeric structures induced CMEC apoptosis, whereas fibrillar aggregates increased BBB permeability without apoptotic effects. Interestingly, all Aβ species tested inhibited angiogenesis in vitro. These data link the biological effects of the heterogeneous Aβ peptides to their primary structure and aggregation, strongly suggesting that the composition of amyloid deposits influences clinical aspects of the AD vascular pathology. As the presence of predominant oligomeric structures in proximity of the vessel walls may lead to CMEC death and induction of microhemorrhages, fibrillar amyloid is likely responsible for increased BBB permeability and associated neurovascular dysfunction. These results have the potential to unveil more specific therapeutic targets and clarify the multifactorial nature of AD.

## Graphical abstract

This study demonstrates that full length Aβ peptides, truncated Aβ fragments, and vasculotropic Aβ variants, differentially impact microvascular endothelial cell function, in relation to their aggregation properties. Understanding these effects on separate but interconnected cerebrovascular degeneration pathways, such as BBB permeability, EC apoptosis and angiogenesis, will provide new insights into the pathology and different clinical subtypes of AD and vascular dementia.

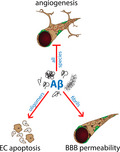

## 1 INTRODUCTION

Alzheimer's disease (AD) is the most common form of dementia, and its incidence is expected to triple by 2050. This progressive neurodegenerative disorder is neuropathologically characterized by the progressive buildup of insoluble amyloid‐β (Aβ) plaques in the brain parenchyma and the intraneuronal accumulation of neurofibrillary tangles (NFT) composed of hyperphosphorylated tau protein. Although it remains unclear what primarily triggers and drives the progression of AD, different lines of investigation point to a central role of Aβ and support the relevance of oligomeric conformations of the peptide in the disease pathogenesis (Selkoe & Hardy, 2016).

The mechanisms leading to the development and progression of AD are complex and likely involve different cellular pathways. Emerging data indicate that reduced brain Aβ clearance—particularly in the elderly—plays a critical role in amyloid formation and AD pathogenesis. Soluble Aβ is cleared from the brain across the vascular endothelium, transported with the bulk flow of interstitial fluid (ISF) into the cerebrospinal fluid (CSF) through the choroid plexus epithelium (McIntee et al., 2016), taken up by microglia, astrocytes, and perivascular macrophages (Wilcock et al., 2004), and removed by glymphatic drainage together with waste products in the ISF. As there are no conventional lymphatic vessels in the brain, the flow of ISF along basement membranes in capillary and artery walls, known as Intramural Peri‐Arterial Drainage (IPAD) plays also a crucial role. During aging, as many of the clearing mechanisms regulating Aβ homeostasis fail or decrease in their efficiency (Bakker et al., 2016), Aβ aggregates accumulate and deposit in the brain parenchyma and around the cerebral vessels as cerebral amyloid angiopathy (CAA). CAA is present in over 80% of AD patients (Arvanitakis et al., 2011) and in as much as 50% of non‐AD elderly over 80 years old. As more Aβ is deposited, arterial walls are disrupted and smooth muscle cells are progressively replaced by Aβ deposits, leading to further impairment of IPAD, microvascular and endothelial cell (EC) dysfunction, and ultimately death (Fossati et al., 2010, 2012; Ghiso et al., 2014; Parodi‐Rullan et al., 2019). The clinical manifestations of CAA are characterized by cerebral hemorrhages, ischemia, hypoperfusion, and cognitive impairment. Although the exact mechanisms leading to vascular Aβ accumulation are still not fully clear, impaired brain Aβ efflux, cerebrovascular (CV) pathology, and cardiovascular risk factors, are likely contributors to vascular amyloid deposition which, in turn, further exacerbates vessel dysfunction and neurodegeneration (Snyder et al., 2015).

Mounting evidence continues to highlight the contribution of pathological changes in vessel hemodynamics, angiogenesis, vascular cell function, and blood‐brain barrier (BBB) permeability to neurodegeneration and cognitive impairment. Amyloid‐associated endothelial tight junction (TJ) pathology, characterized by the loss of TJ proteins and BBB leakage, has been demonstrated as significant contributors to the pathogenesis of human AD and CAA (Nation et al., 2019; Yamazaki et al., 2019), characteristics recapitulated in cellular and animal models of the disease. While there is general consensus regarding the detrimental effect of amyloid on vascular cell toxicity, its contribution to the dysregulation of vascular tone, induction of vascular inflammation, weakening of the BBB, and its impact on cerebral angiogenesis is not well understood. Despite some reports of marked reductions in vascular density and reduced expression of several markers of angiogenesis in the AD brains, other studies have reported increased vascular density within the AD hippocampus, with increase in neo‐angiogenic vessels (Govindpani et al., 2019). This opposing information on the effect of Aβ on microvascular angiogenesis also extends to studies in cell culture and transgenic models, with some reports pointing to inhibited or pathologically defective neovascularization, while others support amyloid‐associated promotion of neo‐angiogenesis and hypervascularity (Biron et al., 2011; Paris et al., 2004; Solito et al., 2009).

Aβ40 and Aβ42 peptides are formed from the proteolytic cleavage of amyloid precursor protein (APP) by the amyloidogenic β‐secretase (BACE‐1) and γ‐secretase. Several APP mutations have been identified that are associated with familial early‐onset amyloidosis. Hereditary cerebral hemorrhage with amyloidosis Dutch type is an example of a well characterized APP mutation, which occurs when glutamate is replaced by glutamine as a result of a point mutation in codon 693 of the APP gene (position 22 of Aβ, Aβ40‐Q22).

Mutation carriers suffer from cerebral hemorrhages and stroke with a mean onset during the 5th decade of life, amyloid accumulation in the brain vasculature and dementia, leading to death by the 6th decade of life (Levy et al., 1990). The Dutch mutation results in a severely toxic and vasculotropic Aβ peptide in comparison with its wild‐type Aβ40 counterpart, likely reflecting the peptide's increased aggregation propensity and tendency for accelerated formation of oligomeric species (Davis & Van Nostrand, 1996; Fossati et al., 2010). In addition to the presence of mutations in the APP gene, post‐translational modifications can also result in peptides with varying aggregation and toxic properties. Among them, the most studied are cyclation of N‐terminal glutamates to form pyroglutamate, isomerization of aspartic residues, methionine oxidation, and enzymatic truncations of the carboxyl and amino‐terminal ends of Aβ (Cabrera et al., 2018; Fossati et al., 2013).

Over the past decades, the presence of various truncated Aβ peptides in the brain, derived from Aβ40/Aβ42 and expressing different N‐ and C‐terminal truncations, has been increasingly reported (Masters et al., 1985; Saido et al., 1996). The N‐terminal truncated peptide Aβ4–42, along with Aβ42 and Aβ40, is one of the most abundant components of fibrillar deposits in the AD brain, as demonstrated by biochemical and mass spectrometry analyses (Masters et al., 1985; Portelius et al., 2010; Rosen et al., 2016; Wildburger et al., 2017). Truncation of the first three amino acids of Aβ, generating Aβ4‐x species, has been shown to increase the Aβ aggregation profile toward the formation of high molecular weight (HMW) oligomers and fibrils, increasing the peptide's neurotoxicity (Bouter et al., 2013; Cabrera et al., 2018). Consisting with these enhanced fibrillogenic properties, Aβ4–42 has been reported to mostly localize to fibrillar Thioflavin and Congo red (+) lesions at the core of parenchymal plaques and in vascular deposits in both, AD and Down syndrome patients, as well as in a number of APP transgenic models including the widely studied Tg2576 and APP/PS1 (Cabrera et al., 2018; Masters et al., 1985; Wirths et al., 2017; Zampar et al., 2020). Notably, transgenic mice specifically expressing Aβ4–42 developed massive CA1 pyramidal neuron loss in the hippocampus that correlates with age‐dependent memory deficits (Bouter et al., 2013).

Contrasting with the biophysical properties of N‐terminally truncated fragments at Phe4, C‐terminal cleavage of Aβ by a number of brain resident enzymes—among them, BACE‐1 and the matrix metalloproteases (MMP) 2 and 9 (Hernandez‐Guillamon et al., 2015; Shi et al., 2003)—results in the formation of more soluble fragments that are less prone to aggregation in in vivo and in vitro models (Cabrera et al., 2018; Hernandez‐Guillamon et al., 2015). Among these truncated species, Aβ1–34 has been consistently identified as a normal component of the CSF Aβ peptidome and can be easily retrieved from brain deposits with the aid of common physiologic buffers (Cabrera et al., 2018; McIntee et al., 2016; Rostagno et al., 2018), it lacks toxicity (Hernandez‐Guillamon et al., 2015), and it has been suggested that it might even have a neuroprotective role (Caillava et al., 2014). In fact, additional C‐terminal truncations that generate shorter Aβ species, such as 1–16, which also exhibit poor aggregation propensity and lack of cytotoxicy (Hernandez‐Guillamon et al., 2015), further suggest that C‐terminal degradations enhance brain clearance mechanisms and are not associated with the amyloidogenic process.

Despite numerous studies focusing on Aβ, the relevance of the N‐ and C‐terminal truncated species for the pathogenesis of AD remains largely understudied. Although some reports have focused on the roles of truncated species in the context of neuronal cell death as well as in their topographical accumulation within parenchymal and vascular deposits (Bouter et al., 2013; Cabrera et al., 2018; Wirths et al., 2017), to our knowledge the contribution of these truncated forms to the induction of CV pathology, as well as their role in the mechanisms of disease pathogenesis in AD and CAA, remains unknown. Based on the recently recognized role of vascular dysfunction as an early pathological feature of AD (Govindpani et al., 2019; Ishii & Iadecola, 2020), and on the importance of the BBB in regulating brain health and maintaining cerebral homeostasis, this study focuses, for the first time, on understanding the role of different amyloid species and Aβ catabolic fragments on cerebral EC death, BBB permeability, and angiogenesis.

## 2 RESULTS

### 2.1 Effects of full‐length Aβ peptides, N‐ and C‐terminal truncated derivatives and the Q22 Dutch mutation on endothelial cell death

**FIGURE 1:**
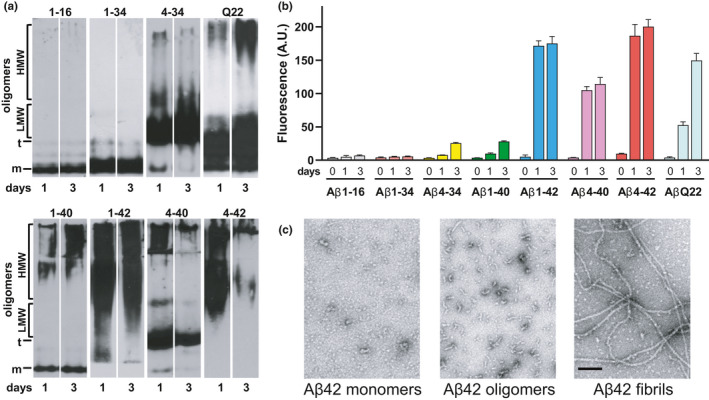
Biophysical and structural analysis of Aβ species. Structural properties of full‐length, N‐and C‐terminal truncated species and the Aβ40‐Q22 mutant, pre‐treated in HFIP and reconstituted in physiologic salt concentration containing buffer were monitored by native gel electrophoresis/Western blot and Thioflavin‐T binding for up to 3 days. (a) Comparative oligomerization profiles after 1‐ and 3‐days incubation assessed by 5%–20% non‐SDS electrophoresis followed by Western blot analysis probed with a 50:50 mixture of anti‐Aβ monoclonal antibodies 4G8 and 6E10. (b) Fluorescence evaluation of Thioflavin‐T binding to the respective synthetic homologues (50 μM) either freshly reconstituted (time 0) or after 1‐ and 3‐day incubation. Results are expressed in arbitrary units (A.U.) and represent the mean ± SEM of three independent experiments after subtraction of blank levels. (c) Structural assessment of Aβ1–42 oligomers and fibrils in comparison with the non‐aggregated counterpart prepared as described in Materials and Methods and visualized by EM after negative staining with uranyl acetate. Bar represents 100 nm.

Our previous work demonstrated differences in the aggregation properties and oligomerization kinetics among the full‐length peptides Aβ40/42, their N‐ or C‐terminally truncated forms, and Aβ peptides bearing familial mutations associated with vascular toxicity, such as the Dutch mutant (Cabrera et al., 2018; Fossati et al., 2010). These studies demonstrated that the structural characteristics of the different peptides correlated with amino acid sequence variations and the length of the peptide chain, showing that truncations at the C‐termini, such as those generating Aβ 1–34 and 1–16, lead to the production of soluble fragments with no propensity for aggregation (Cabrera et al., 2018; Hernandez‐Guillamon et al., 2015) and Figure 1a and b). On the contrary, N‐terminal truncations at position 4, generating Aβ 4–40 or 4–42, induce the formation of poorly soluble, aggregation‐prone peptides with higher amyloidogenic propensities than their full‐length counterparts (Cabrera et al., 2018 and Figure 1a and b). Additional truncations at position 34 seem to partially abrogate the amyloidogenic characteristics; the double truncated 4–34 forms low molecular weight (LMW) oligomeric assemblies while precluding fibrillization in the time frame of our studies. The presence of mutations associated with the development of CAA and early‐onset CV pathology, such as the Q22 mutation, elicits increased aggregation propensity in comparison to its wild‐type counterpart, although the aggregation species formed by the mutated peptides are a mixture of HMW and LMW oligomeric assemblies (Cabrera et al., 2018; Fossati et al., 2010).

**FIGURE 2:**
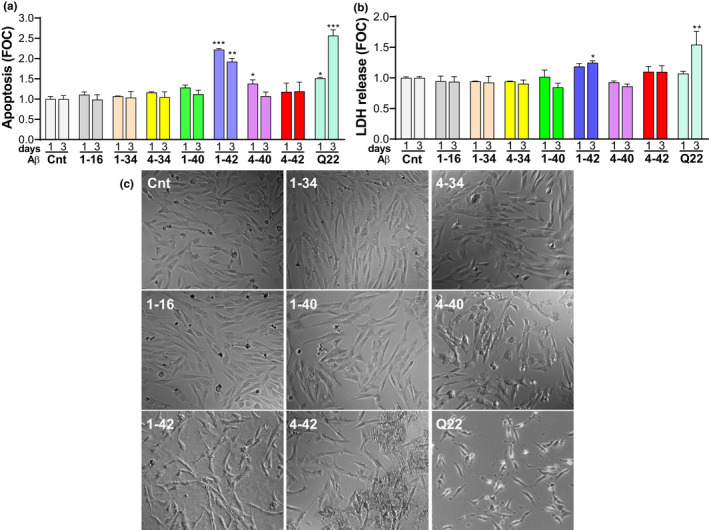
Induction of CMEC apoptosis and necrosis by full length, truncated, or mutated Aβ peptides. (a) Measurement of apoptotic cell death after treatment with 10 µM of each Aβ peptide or fragment for 1 and 3 days in CMEC. Apoptotic cell death was assessed as formation of fragmented nucleosomes using the Cell Death Detection ELISA kit (Roche). (b) Necrotic cell death, measured as amount of LDH release, after treatment of CMEC with 10 µM of each Aβ peptide or fragment for 1 and 3 days. LDH activity was assessed using the Cytotoxicity Detection Kit^PLUS^ (Roche) as the production of red formazan from tetrazolium salt, an NADH‐dependent reaction. (c) Representative images of contrast phase microscopy of CMEC after 24‐h treatment with 10 µM of each Aβ peptide or fragment. Cnt indicates untreated control group. Graphs are representative of 3 individual experiments of 2 replicates per group. *p < 0.05, **p < 0.01, and ***p < 0.001 versus Cnt.

To better understand the implications of the presence of all these peptides in the AD and CAA brain as well as the impact of this structural heterogeneity on the pathophysiology of CV deposits, we examined the effects of full length, truncated, or mutated Aβ peptides on CMEC death, BBB permeability, and angiogenesis. We first evaluated CMEC death, differentiating between apoptosis (measured as the number of fragmented nucleosomes, Figure 2a), and necrosis (evaluated as LDH release, Figure 2b). Notably, peptides which do not aggregate over time and remain mostly in monomeric form, such as Aβ fragments 1–16 and 1–34, as well as those that primarily form LMW oligomers (4–34) or exhibit low propensity to form HMW oligomers within our experimental window (4–34 and 1–40) do not cause CMEC apoptosis after 1 or 3 day treatment with 10 µM peptide concentration. In contrast, Aβ1–42, which is known to rapidly form LMW and HMW oligomers and eventually fibrils within a few hours after solubilization in cell culture medium, as well as the Q22 mutant, which also forms LMW and HMW oligomers (Figure 1a and b), caused apoptosis at both 1 and 3 days. Both, Aβ1–42 and Q22 were also capable of inducing secondary necrosis after 3 days treatment (Figure 2b), likely as a result of the breakdown of apoptotic cells, with the Dutch variant being more pro‐apoptotic than Aβ1–42 at 3 days, correlating with a longer permanence of oligomeric species in solution (Fossati et al., 2010). Similarly, the N‐terminally truncated peptide Aβ4–40, which aggregates more efficiently than the Aβ1–40 counterpart, forming LMW and HMW oligomeric species during the first few hours in cell medium (Cabrera et al., 2018), also caused significantly higher levels of apoptosis than Aβ1–40 at 1 day. Interestingly, the peptide with the highest aggregation propensity, Aβ4–42, which aggregates very quickly and forms mostly fibrillar clumps in physiological solutions (Bouter et al., 2013; Cabrera et al., 2018), did not induce any significant form of cell death, apoptosis nor necrosis, following 1 or 3 days of treatment (Figure 2a and b), in contrast to what has been reported in neurons (Bouter et al., 2013). Evaluation of the cell morphology by contrast phase (CP) microscopy 24 h after peptide challenge (Figure 2c) confirmed the appearance of cell toxicity phenotypes, such as cell shrinkage and derangement, in cells incubated with Aβ1–42 or Q22, as well as, to a lower extent, with Aβ4–40. Importantly, despite the absence of apoptotic or necrotic characteristics in Aβ4–42‐treated cells, the CP images illustrate an impaired growth compared with untreated cells, whereas fibrillar aggregates are observed deposited in parts of the culture well. It is possible that these fibrillar aggregates, while not triggering apoptosis, may cause damage to membrane structures and impair the ability of the cells to absorb nutrients, hindering BBB function. Therefore, we proceeded to analyze how the different peptides affected the BBB functionality.

### 2.2 Effects of intact, truncated, and mutated Aβ peptides on BBB resistance

**FIGURE 3:**
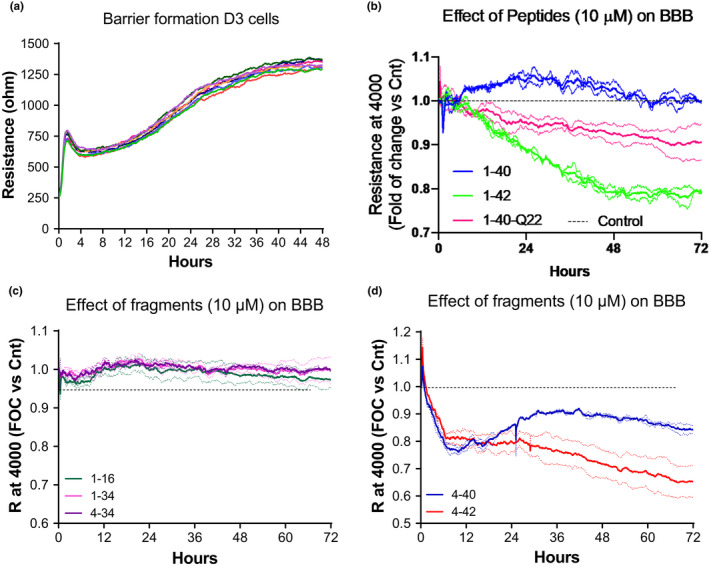
Effects of full length, truncated, or mutated Aβ peptides on CMEC barrier permeability. CMEC barrier was assessed by monitoring TEER with the ECIS Zθ system (Applied Biophysics) which measures membrane resistance and capacitance in real time. (a) Formation of BBB‐like cell monolayer as a function of elevated and stable resistance after about 48 h on the electrodes. (b–d) Barrier permeability, assessed for 72 h as a reduction in resistance, after treatment with 10 µM Aβ40, Aβ42, Aβ40‐Q22 (b); 1–16, 1–34, 4–34 (c); 4–40, 4–42 (d), all compared to untreated control (dashed line). Graphs represent at least 3 individual experiments of 2 replicates per group.

Trans‐endothelial electrical resistance (TEER) of EC monolayers was monitored in real time using the ECIS Zθ system in an effort to evaluate whether the action of Aβ peptides, their fragments, or the Dutch mutant on BBB function and permeability mirror the cell toxicity effects described above or if they are independently regulated. CMECs were seeded on multi‐well plates containing 40 electrodes/well and connected to the ECIS Zθ instrument, which measures membrane resistance and capacitance in real time. The cells formed monolayers, reaching elevated and stable electrical resistance 48 h after seeding, modeling the properties of the BBB, as illustrated in Figure 3a. After reaching TEER plateau, cells were treated with the different peptides and their effect on BBB permeability was monitored as reduction in TEER (compared to untreated cells) for the following 72 h (Figure 3b–d). Peptides that remained mostly monomeric or exhibit low propensity to form HMW oligomers during the first 2–3 days in culture medium—Aβ fragments 1–16, 1–34, and 4–34 as well as Aβ1–40—did not cause any decrease in resistance during the first 72 h (Figure 3b and c). In addition, species forming a mixture of LMW and HMW oligomers, like the Q22 peptide, had limited effects on BBB permeability (Figure 3b). In contrast, peptides that rapidly aggregate into fibrillary conformations, such as Aβ1–42 and 4–42 (Figure 3b and d), had stronger effects on inducing loss of BBB resistance. Indeed, the decrease in TEER was most evident with peptides that showed higher tendency to form fibrils. Among them, the effect of Aβ4–42 was the highest observed. Notably, the effect of Aβ4–40 on TEER was more pronounced than that of Aβ1–40, but somehow comparable with that of the Dutch mutant. The ability of some of the peptides with higher fibrillization propensity, such as Aβ4–42 to modify BBB resistance without compromising cell viability, suggests that fibrillar species may preferentially cause BBB permeability alterations, while oligomeric species, particularly those of HMW, are likely to have more potent effects on EC apoptosis.

### 2.3 Effects of Aβ42 oligomers versus fibrils on apoptosis and BBB permeability

**FIGURE 4:**
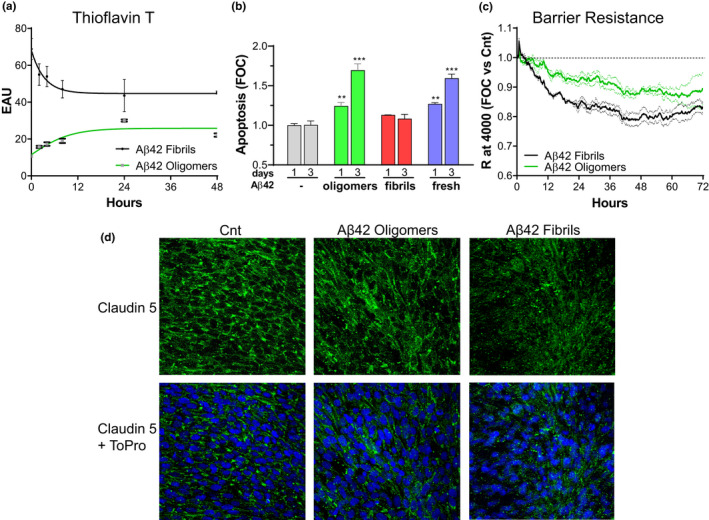
Differential effects of oligomeric and fibrillar Aβ42 on CMEC death and BBB permeability. (a) Thioflavin‐T binding of oligomeric and fibrillar Aβ42 revealing the stability of these species for days after solubilization in cell medium. (b) Apoptotic cell death after treatment of CMEC with 10 µM of freshly solubilized, oligomeric, and fibrillar Aβ42 for 1 and 3 days. (c) Barrier resistance, assessed for 72 h after treatment with 10 µM of oligomeric or fibrillar Aβ42 and compared to untreated control (dashed line). (d) Representative images of the TJ protein claudin‐5 (green) in CMEC monolayers after 1 day of treatment with 10 µM of freshly solubilized, oligomeric, or fibrillar Aβ42. Cell nuclei are counterstained using ToPro nuclear staining (blue). Cnt indicates untreated control monolayers. Images and graphs are representative of 3 individual experiments of 2 replicates per group. *p < 0.05, **p < 0.01, and ***p < 0.001 versus Cnt.

To evaluate whether oligomeric and fibrillar assemblies exhibited differential effects on EC death and barrier permeability, we prepared separate solutions enriched in oligomeric and fibrillar Aβ42 aggregates, as described in the experimental procedures, following well‐established protocols designed to obtain oligomeric or fibrillar preparations (Solesio et al., 2018; Stine et al., 2003). The characteristics and composition of the oligomeric and fibrillar assemblies formed under our experimental conditions were confirmed by electron microscopy analysis, as illustrated in Figure 1c. Monitoring the Thioflavin‐T binding capacity of these species over time demonstrated that they remain stable for days after solubilization in cell culture medium (Figure 4a). As expected, fibrillar preparations equilibrated in EBM‐2 at 10 µM final concentration, exhibited higher fluorescence intensity upon binding to Thioflavin T with levels over 60 EAUs. The fluorescence slightly decreased as the fibrils equilibrated in solution, reaching a plateau after about 12 h with values above 45 EAUs. Oligomeric preparations, as anticipated, showed lower Thioflavin‐T binding properties than fibrils with values starting at about 15 EAUs, and reaching equilibrium at 20 EAUs after about 12 h. These results confirmed the stability of the oligomeric and fibrillary preparations employed in our assays throughout our experimental timeframe and indicate that both types of assemblies maintain distinct aggregation properties and Thioflavin‐T binding capacities which were clearly distinguishable from each other.

CMECs were separately treated with either freshly solubilized Aβ42 or with preparations enriched in oligomeric and fibrillar assemblies at 10 μM concentration each, monitoring apoptosis for up to 3 days, as illustrated in Figure 4b. Oligomers and freshly solubilized peptides had significantly higher pro‐apoptotic effects than fibrils, both after 1 and 3 days of treatment, although the difference was more pronounced after 3 days, when cells treated with fibrils did not show significant differences in apoptosis compared to untreated cells. Evaluation of apoptosis induction demonstrated that treatment with oligomers and freshly solubilized peptides significantly increased the number of fragmented nucleosomes generated from day 1 to day 3. The stronger effect on EC apoptosis of Aβ oligomers compared to fibrils of the same peptide is consistent with the results shown for Aβ mutants and truncated fragments which demonstrated a correlation between the degree of oligomerization and the ability of the peptides to induce nucleosome formation (Figure 2a). Indeed, based on these results, we expected oligomeric preparations to have a greater effect on apoptosis and anticipated that fibrils are likely to exhibit more potent effects on BBB permeability, similarly to the effect exhibited by the highly fibrillogenic Aβ4–42 peptide (Figure 3). For further confirmation, we treated CMEC monolayers with Aβ1–42 oligomeric and fibrillary preparations and monitored their effect on BBB resistance in the ECIS system (Figure 4c). As expected, Aβ42 oligomers caused a small decrease in TEER similar to the Q22 mutant. In contrast, Aβ42 fibrils had a more powerful effect on TEER, further confirming that fibrils impact more BBB resistance compared with oligomeric counterparts. In spite of the clear difference in the cellular responses yielded by incubation with oligomer‐ or fibril‐enriched preparations, it should be noted that the potential contribution of other minor aggregation species, albeit present in minimal amounts in the respective peptide preparations, cannot be completely ruled out.

As a further validation of the differential effects of oligomers and fibrils on BBB permeability and function, we assessed the expression and localization of the TJ protein claudin‐5 in CMEC monolayers. As expected, claudin‐5 staining, illustrated by the green fluorescence in Figure 4d, was mostly localized to the cell membranes in untreated cell monolayers. Aβ1–42 oligomers, applied after the cells had been maintained in confluent monolayers for 5 days, and incubated for 1 day, slightly affected claudin‐5 localization, revealing a less clear membrane staining and some apparent cytoplasmic localization. Fibrils, on the other hand and as anticipated, exhibited a stronger effect on claudin‐5 membrane distribution. Cell monolayers incubated 1 day with Aβ1–42 fibrils showed an almost complete loss of claudin‐5 membrane localization, with a mostly cytoplasmic distribution of the junction proteins. Cell loss in the confluent monolayers induced by peptide treatment was not evident, as confirmed by the blue fluorescence signal of the ToPro nuclear staining.

### 2.4 Effects of Aβ peptides, truncated fragments, and the Dutch mutant on angiogenesis

**FIGURE 5:**
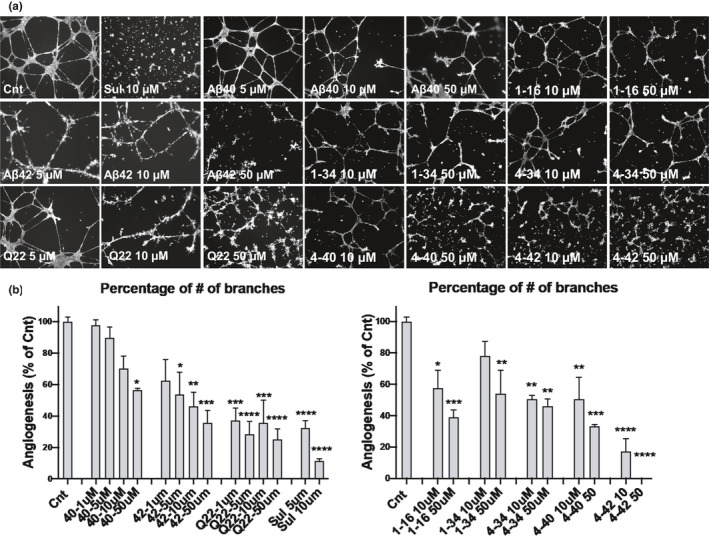
Inhibition of angiogenesis by Aβ peptides and fragments. Inhibition of angiogenesis was assessed using the Millipore's Millicell μ‐Angiogenesis Inhibition Assay. CMECs were treated for 24 h with the different amyloid species and monitored by fluorescent staining with 50 μM Calcein AM. (a) Representative images of the effects of Aβ on new‐vessel formation. (b) Number of capillary branches, indicative of angiogenesis, expressed as percent change from untreated controls in each individual experiment. For each experiment capillary branches were counted from 4 randomized images for each treatment. Cnt indicates the untreated control group. Sulforaphane (Sul, 5 or 10 μM) was used as positive control for angiogenesis inhibition. Graphs represent 3 individual experiments of 2 replicates per group. *p < 0.05, **p < 0.01, and ***p < 0.001 versus Cnt.

Angiogenesis has been reported as a repair system in conditions associated with brain trauma and neurodegenerative disorders (Zhang et al., 2018), including AD and CAA (Merkulova‐Rainon et al., 2018). In view of the observed EC death and BBB dysfunction induced by full length and truncated Aβ peptides, it is possible that the brain vasculature may attempt to repair these injuries through neo‐angiogenesis. While multiple reports have shown AD‐associated angiogenic alterations, which have been recapitulated by Aβ‐challenge in in vitro experiments (Koster et al., 2016; Merkulova‐Rainon et al., 2018), the importance of Aβ catabolic fragmentation or familial mutations in these events has not been previously studied. In the current work, we tested how C‐ and N‐terminally truncated fragments as well as full‐length Aβ40, Aβ42, and the Q22 variant impact new‐vessel formation using the Millipore angiogenesis inhibition assay (Figure 5), applying Sulforaphane as a positive inhibitory control.

We observed that all peptides and fragments, including the most soluble ones, have detrimental effects on the formation of vessels, independently from their effects on EC death or BBB permeability. In particular, the catabolic fragments 1–16, 1–34, and 4–34, which do not form HMW oligomers under our experimental conditions, and do not induce apoptosis, necrosis, or reduction in TEER, were able to inhibit angiogenesis in a dose‐dependent manner, reaching significant effects at 10 and 50 μM concentrations (Figure 5a and b‐right panel). The inhibitory effect of Aβ1–42 on angiogenesis was higher than that of Aβ1–40 and significant at 5 μM, while 1 μM Q22 already inhibited angiogenesis to levels below 50% of untreated controls. Interestingly, Aβ4–40 affected angiogenesis more robustly than the Aβ1–40 counterpart. The Aβ4–42 truncated peptide was the most potent angiogenesis inhibitor, decreasing the number of branches at a 10 μM concentration to 20% of the untreated controls, almost comparable to the potent inhibitor sulforaphane. This potent anti‐angiogenic capacity of Aβ4–42 contrasts with its inability to cause apoptosis or necrosis at the same concentrations (shown in Figure 2). It should be mentioned that although for some of the peptides such as Q22 or Aβ42, the inhibition of angiogenesis could be partially attributed to their ability to induce apoptosis, this is unlikely the case for low concentrations of the peptides or for other peptides, such as 4–42, which do not induce apoptosis in spite of causing severe angiogenesis inhibition.

Overall, our results indicate that all Aβ peptides, including monomeric forms, if present in the perivascular spaces or in vascular deposits in sufficient concentrations, can impair neo‐angiogenesis, a much‐needed repair mechanism for injured vessels in the AD and CAA brain. The inhibitory effect on angiogenesis exerted by the different Aβ peptides and fragments appear to be in part, but not exclusively, related to their aggregation propensity since monomeric forms of peptides lacking ability to oligomerize are also able to prevent EC angiogenesis. The dichotomy between the ability of monomeric peptides to cause the angiogenesis deficits and their failure to trigger EC death and BBB permeability alterations suggests that Aβ participation in these pathways is likely mediated by independent cellular mechanisms.

## 3 DISCUSSION

During the past decades, the presence of 40 and 42 amino acid‐long Aβ peptides, their catabolic fragments, and mutated forms of the peptides, has been extensively recognized in AD and CAA brains (Cabrera et al., 2018; Masters et al., 1985; Saido et al., 1996). The complexity of amyloid species present in parenchymal plaques and/or in brain vascular deposits—with different biophysical characteristics and aggregation propensities—can influence the overall pathophysiology and clinical manifestations of the disease. This study sought to understand how different truncated or mutated species of Aβ, as well as the canonical full‐length Aβ40/42, affect the BBB functionality, focusing on multiple physiological aspects of CV dysfunction, including EC viability, monolayer permeability, and angiogenesis, and how the divergent biological actions of these peptides are associated with the aggregation/fibrillization properties. Indeed, the current work clearly demonstrates that Aβ peptides and truncated fragments, as well as different types of aggregates, exhibit distinct toxic properties and induce dissimilar types of vascular dysfunction.

We first examined the ability of Aβ peptides, their catabolic fragments, and the vasculotropic Dutch mutant, to induce apoptotic and necrotic EC death. It is amply recognized that differences in aggregation characteristics have high impact on the toxic properties of different Aβ molecules (Fossati et al., 2010, 2012; Stine et al., 2003). Along this line, the current results demonstrate that species that form HMW and LMW oligomers such as Aβ42, Q22, and Aβ4–40 induce EC apoptosis and secondary necrosis under our experimental conditions (Figures 1a and b and 2a and b) (Cabrera et al., 2018; Fossati et al., 2010, 2012). In contrast, Aβ peptides and truncated fragments that remain mostly monomeric, as Aβ 1–16 and 1–34, as well as those that show slow aggregation properties as Aβ40, or those that quickly form fibrillar structures as 4–42, are all unable to induce apoptotic or necrotic cell death. These results were further validated by the use of pre‐aggregated Aβ42 under different experimental conditions that lead to the formation of either oligomeric‐ or fibril‐enriched structures (Figure 1c) (Stine et al., 2003). In these experiments, apoptosis was induced by freshly solubilized and pre‐formed oligomeric Aβ42 species, which form HMW oligomers by 3 h in culture conditions (Cabrera et al., 2018; Fossati et al., 2010), but not by fibrillar forms of the peptide (Figure 4b). Similarly, previous studies have shown that oligomers are among the most toxic Aβ species (Benilova et al., 2012; Fossati et al., 2010), inhibiting neuronal viability 10 folds more than fibrils. Additionally, and in agreement with these in vitro data, several groups have demonstrated that, in AD cases, soluble Aβ species better correlate with cognitive function than the number of plaques (composed of fibrillar Aβ) (Cleary et al., 2005). However, the mechanisms by which oligomeric structures exert their toxic effects are still unclear, with some studies indicating that they may form ion‐like channel structures causing subsequent calcium dysregulation and alterations of cell homeostasis (Quist et al., 2005) or disrupt membrane structure in a detergent‐like manner (Bode et al., 2019). This effect is not observed in the case of monomeric and fibrillar Aβ conformations although fibrils have been shown to laterally associate and embed into the upper leaflet of the membrane bilayer (Bode et al., 2019). We have previously demonstrated that Aβ oligomers serve as signaling molecules by binding and activating the TRAIL death receptors DR4/5 and initiating apoptotic cell death in CMEC (Fossati et al., 2010, 2012). It is conceivable that oligomeric structures of the different Aβ peptides studied herein may also be capable of inducing CMEC death through the same mechanisms, binding to TRAIL death receptors and activating the extrinsic apoptotic pathway. The contribution of other alternative mechanisms, such as a direct effect of oligomers on membrane structures or the induction of independent detrimental intracellular effects, cannot be ruled out as contributors to the deleterious consequences of these peptides on cell viability.

Brain homeostasis is maintained through a tight regulation of permeability barriers between the cerebrospinal fluid (CSF) and ISF—brain CSF barrier—and between the brain and the circulating blood through formation of the BBB, which is characterized by the presence of TJ and adherence junctions in EC. An increase in BBB permeability has been associated with cognitive decline (Nation et al., 2019) and has been observed prior to the development of cognitive dysfunction, preceding amyloid, and tau brain accumulation (Ishii & Iadecola, 2020; Nation et al., 2019). Different mechanisms have been shown to contribute to the dysregulation of BBB function. Alterations of BBB permeability have been attributed to EC loss of TJ and adherence junctions, mechanisms facilitated by activation of cyclophilin A (CypA) and MMP‐9 through an APOE4‐mediated increase in soluble platelet‐derived growth factor β (sPDGFβ), a marker of pericyte injury (Ishii & Iadecola, 2020; Montagne et al., 2020; Nation et al., 2019). In addition, inflammation, as well as the direct actions of Aβ on EC functionality (Yamazaki et al., 2019), has also been demonstrated as important contributors to BBB dysfunction.

In the current study, we focused on understanding the differential impact of Aβ peptides and truncated fragments on BBB permeability and TJ expression evaluating whether these effects correlate with the aggregation/fibrillization propensity of the different Aβ species. Our results demonstrate that Aβ peptides adopting predominantly fibrillar conformations such as Aβ42 and 4–42 exert the most detrimental effects on BBB permeability (Figure 3b and d). On the other hand, peptides that either remain mostly monomeric as Aβ 1–16 and 1–34, or form LMW oligomers such as Aβ40 and 4–34 (Figure 1a and b) (Cabrera et al., 2018) have little to no effect on EC monolayer TEER (Figure 3b and c), while oligomeric and protofibrillar species such as those formed by the Q22 mutant (Fossati et al., 2010) have lower effects than fibrils in inducing BBB permeability (Figure 3). Further validation through the use of pre‐formed enriched preparations of Aβ42 oligomers and fibrils (Figure 1c) corroborated that fibrillar assemblies increase BBB permeability to a greater extent than oligomers (Figure 4c). In turn, this permeability changes correlated with the decrease in the expression and membrane distribution of the TJ protein claudin‐5 (Figure 4d). Our findings are supported by work from other groups that reported loss of TJ in human post‐mortem brains and in co‐culture systems of iPSC‐derived neurons and CMECs (Yamazaki et al., 2019). Importantly, as BBB integrity is compromised and neurovascular damage is triggered, amyloid deposition increases and clearance is impaired leading to the exacerbation of vascular pathology in the AD and CAA brain, with further worsening of neurodegeneration and creating a vicious cycle that conducts to a progressive increase in cognitive decline.

One of the mechanisms by which the brain responds to vascular compromise is through the induction of angiogenesis, a process that plays significant roles in neurovascular regeneration and maintenance of brain homeostasis. Defective angiogenesis has been reported in conditions of trauma and neurodegeneration such as those observed in AD and CAA (Biron et al., 2011) while in contrast, neovascularization and concomitant increase in microvessel density have been shown to serve as a repair system capable of improving cognition in APP transgenic models (Zhang et al., 2018). Different cellular mechanisms have been shown to affect endothelial angiogenesis. The presence of hypoxia frequently associated with conditions of cerebral hypoperfusion as well as the increased levels of pro‐inflammatory cytokines observed during BBB breakdown lead to the increased production of vascular endothelial growth factor (VEGF), epidermal growth factor (EGF), and fibroblast growth factor (FGF), all of which have—in turn—been shown to induce angiogenesis through the promotion of EC growth and enhanced cell division. Under physiological conditions, angiogenesis and neovascularization constitute responses to impaired oxygen and nutrient delivery and as such, should have the capacity to act as important repair mechanisms in conditions such as AD and CAA. Conflictingly, Aβ, an important element in AD pathogenesis, is a negative regulator of angiogenesis (Merkulova‐Rainon et al., 2018; Paris et al., 2004; Solito et al., 2009).

In the current work, we demonstrate that supra‐physiological concentrations (in the low μM range), likely to be present in CAA deposits, of all Aβ peptides and fragments tested negatively affect neo‐angiogenesis in human CMEC, independently of their capacity to elicit cell toxicity or induce BBB permeability alterations (Figure 5). Measuring the release of pro‐ or antiangiogenic factors induced by the different peptides as well as evaluating multiple endothelial and junction‐protein markers under different conditions in vitro and in vivo is a natural next step in the development of this line of research and is the subject of current work in our laboratory. Although both increased and defective angiogenesis have been previously reported in AD (Biron et al., 2011; Cantara et al., 2004; Koster et al., 2016), differences in the Aβ concentrations and peptide composition, as well as the dissimilar aggregation state of the amyloid deposits within the vessel microenvironment could account for the discrepancies among the different studies (Biron et al., 2011; Cantara et al., 2004). Supporting this notion, our data clearly demonstrates a dose‐dependent inhibition of angiogenesis (Figure 5), suggesting that higher Aβ concentrations, such as those associated with dense CAA deposits, are able to interfere and block pro‐angiogenic stimuli, thereby inhibiting the natural response to injury (Cantara et al., 2004; Paris et al., 2004; Solito et al., 2009). An alternative mechanism for Aβ antiangiogenic properties may be the reported ability of the peptide to compete with pro‐angiogenic factors for receptor binding. Indeed, the overexpression of pro‐angiogenic factors such as EGF and FGF has been shown to counteract the detrimental effects of Aβ (Koster et al., 2016), research that opens up new potential therapeutic approaches.

**FIGURE 6:**
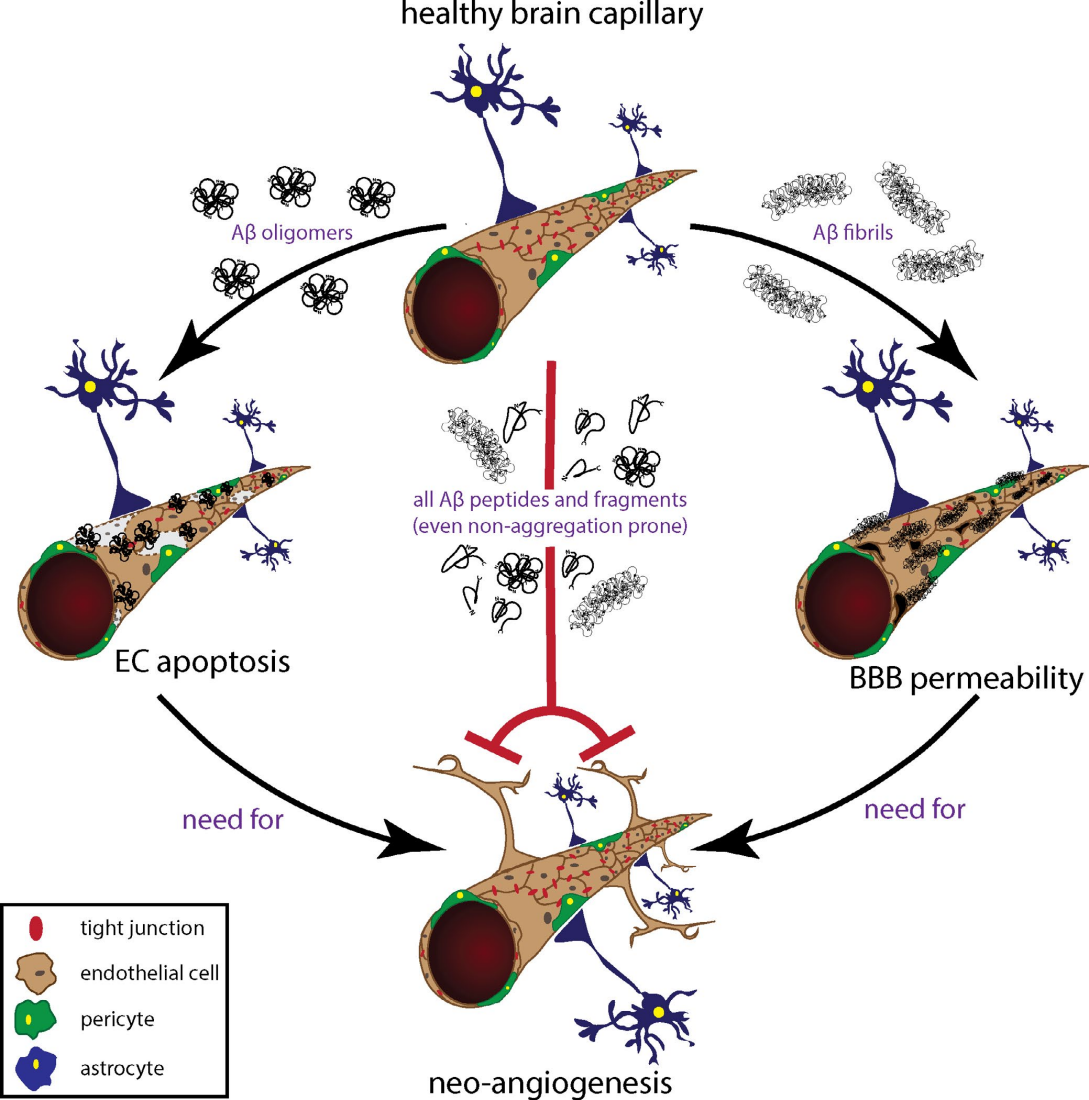
Schematic of proposed model. A healthy brain capillary is represented at the top. The presence of oligomeric Aβ species induces EC death (left arrow), while fibrillary Aβ species increase BBB permeability (right arrow). Damage to the brain capillary due to EC death or BBB dysfunction would need the activation of angiogenesis as a repair mechanism. However, due to the presence of Aβ (all aggregation species and fragments) angiogenesis is inhibited (central red line).

Overall, our data clearly demonstrate that individual Aβ peptides, catabolic truncated fragments, and specific vasculotropic genetic variants elicit different actions on CMEC physiology, which correlate with the aggregation propensities of the different molecules. We demonstrate for the first time that, while CMEC death is mainly mediated by oligomeric Aβ forms, loss of BBB resistance is preferentially mediated by fibrillar Aβ, while all peptides and their fragments, including monomeric forms, are capable of detrimentally affecting angiogenesis if present at high concentrations (Figure 6). Although further studies in in vivo models are warranted to validate the current findings in a more physiological environment, these results support the importance of discriminating the role of specific Aβ peptides and truncated fragments, as well as different types of aggregates, in neurodegeneration. Indeed, the dissimilar aggregation properties and heterogeneous composition of amyloid deposits may directly reflect differences in clinical pathology, with mostly oligomeric vascular accumulations leading to vessel wall cell death and development of microhemorrhages, and fibrillar amyloid deposition responsible for the increase in BBB permeability, with associated inflammation and neurovascular dysfunction. Indeed, the potential role of peptide‐induced inflammation and its contribution to the generation of reactive oxygen species, apoptotic bodies, and pro‐inflammatory cytokines by CMECs, cannot be underestimated. Pathological vascular mechanisms are likely to play an important role in the inflammatory and autoimmune events characterizing some patients with CAA and AD, CAA‐related inflammation (Poli et al., 2020), or patients receiving anti‐Aβ therapeutic antibodies, who may develop amyloid‐related imaging abnormalities (ARIA) (Piazza & Winblad, 2016). Understanding how these separate but interconnected pathways correlate with the onset and progression of AD and CAA, as well as clarifying the specific mechanisms by which each of these vascular pathologies influences neurodegeneration, would certainly provide new insights into the pathological and etiological differences present in the different clinical AD subtypes, and unveil new and more specific therapeutic targets for this multifactorial disorder.

## 4 EXPERIMENTAL PROCEDURES

### 4.1 Aβ peptides

Synthetic full‐length Aβ40 and Aβ42, N‐terminal truncated fragments 4–40, 4–42 and 4–34, C‐terminal truncated fragments 1–16 and 1–34, and the peptide containing the Dutch variant, Aβ40‐Q22, were synthesized using N‐tert‐butyloxycarbonyl chemistry at ERI Amyloid Laboratory, and by Peptide 2.0, as previously described (Cabrera et al., 2018). Peptides were dissolved in hexafluoroisopropanol (HFIP) at a 1 mM concentration, incubated overnight to allow the breakdown of secondary structures and obtain monodisperse preparations, (Fossati et al., 2010) and lyophilized using a Benchtop Freeze Dryer (LABCONCO). Lyophilized peptides were resuspended to a 10 mM concentration in dimethyl sulfoxide (DMSO) and deionized water was added to achieve a final concentration of 1 mM. Prior to the cell culture experimental procedures, peptides were further diluted in endothelial basal media (EBM‐2) media supplemented with 1% fetal bovine serum (FBS).

Pre‐aggregated Aβ42 oligomers and fibrils were prepared from HFIP‐treated and lyophilized peptides as previously reported (Stine et al., 2003). Briefly, fibril‐enriched Aβ42 was prepared by dilution of the lyophilized peptide in DMSO at a concentration of 5 mM followed by further dilution with 10 mM HCl to a 100 μM final concentration and subsequent incubation for 24 h at 37°C. Oligomer‐enriched Aβ42 preparations were generated by dilution of the peptide in DMSO at a concentration of 5 mM, subsequent dilution to 100 μM in ice‐cold EBM‐2 media, and incubation for 24 h at 4°C. Aβ42 oligomers and fibrils were dissolved in EBM‐2 medium to a 10 μM concentration prior to the cell culture experiments or to the assessment of structural stability in vitro.

### 4.2 Thioflavin‐T binding

Aggregation propensity of intact and truncated Aβ species as well as stability of pre‐aggregated Aβ42 oligomeric and fibrillar assemblies was assessed by monitoring thioflavin‐T binding for up to 72 h, as previously described (Cabrera et al., 2018; Fossati et al., 2016).

### 4.3 Electron microscopy

Aβ42 oligomerization and fibrillization were monitored by electron microscopy (EM), as previously described (Fossati et al., 2010). Five microliters of the Aβ preparations were placed onto carbon‐coated 400 mesh Cu/Rh grids (Ted Pella, Inc.) and stained with 1% uranyl acetate in distilled water (Polysciences, Inc.). Stained grids were examined in a Philips CM‐12 transmission electron microscope and photographed with a Gatan (4 k × 2.7 k) digital camera at the Image Core Facility of the Skirball Institute of Biomedical Medicine, NYU School of Medicine (Fossati et al., 2010; Solito et al., 2009).

### 4.4 Native gel electrophoresis and Western Blot analysis

Assessment of peptide aggregation was carried out via native gel electrophoresis in 5%–20% gradient polyacrylamide gels, using 25 mM Tris/glycine buffer, pH 8.8, as running buffer. After separation, proteins were electrotransferred to 0.45 μm nitrocellulose membrane (Hybond‐ECL, GE Healthcare Life Sciences) and visualized with a 50:50 combination of monoclonal anti‐Aβ antibodies 4G8 (epitope: residues Aβ18–22) and 6E10 (epitope: residues Aβ3–8), both from Covance, at a 1:3000 dilution each, followed by incubation with horseradish peroxidase (HRP)‐labeled F(ab’)2 anti‐mouse IgG (1:5000; GE Healthcare) as previously described (Cabrera et al., 2018).

### 4.5 Cell culture

Immortalized human cerebral microvascular endothelial cells (CMEC) were obtained from Babette Weksler (Cornell University). Cells were grown in EBM‐2 (Lonza) and supplemented with growth factors (Hydrocortisone, hFGF‐B, VEGF, R3‐IGF‐1, ascorbic acid, hEGF, and GA‐1000) and 5% FBS and maintained at 37°C in a humidified cell culture incubator under a 5% CO_2_ atmosphere. Cells were visualized and imaged using the EVOS M5000 Imaging System (Thermo Fisher Scientific).

### 4.6 Apoptotic cell death

Apoptotic cell death was assessed as formation of fragmented nucleosomes using the Cell Death Detection ELISA^Plus^ kit (Roche Applied Science) according to the manufacturer's instructions. Briefly, CMEC was seeded and after 24 h treated with 10 µM solutions of the different full‐length Aβ peptides, the truncated Aβ forms, or pre‐aggregated Aβ42 in EBM‐2 media supplemented with 1% FBS. DNA‐histone complexes were measured with Cell Death Detection ELISA^Plus^ at 405 nm using the FlexStation 3 Multi‐Mode Microplate Reader (Molecular Devices).

### 4.7 Lactate dehydrogenase activity

Lactate dehydrogenase (LDH) activity in the cell culture supernatant, indicative of necrosis, was assessed using the Cytotoxicity Detection Kit^PLUS^ (Roche Applied Science) according to the manufacturer's instructions. The conversion of pyruvate to lactate by LDH results in the reduction of NAD^+^ to NADH. The activity of LDH was assessed as the production of red formazan from tetrazolium salt, an NADH‐dependent reaction. Absorbance was measured at 490 nm using the FlexStation 3 Multi‐Mode Microplate Reader (Molecular Devices).

### 4.8 Immunocytochemical imaging of tight junction proteins

Evaluation of the expression and localization of the TJ protein claudin‐5 after Aβ peptide treatment in CMEC cells was evaluated by immunocytochemistry (ICC) as previously described (Fossati et al., 2010). Briefly, cells were plated on collagen‐coated glass chamber slides (Thermo Fisher Scientific) and were grown in EBM‐2 0.25% FBS +bFGF 1:2000 to facilitate the development of tight junctions. After cells were confluent for 5 days, cells were treated for 24 h with 10 μM of Aβ42 oligomers, Aβ42 fibrils, or freshly solubilized Aβ42, washed with PBS, and subsequently fixed for with 4% paraformaldehyde at room temperature. Claudin‐5 was visualized by incubation with anti‐claudin‐5 mouse monoclonal antibody (Invitrogen), followed by an incubation with Alexa Fluor 488‐conjugated anti‐mouse IgG (Invitrogen) and nuclei visualized by counterstaining with To‐pro (Invitrogen). Images were then acquired using Nikon Eclipse Ti inverted fluorescence microscope with deconvolution. Specificity of the antibody detection was assessed by omission of the primary antibody during the ICC procedure.

### 4.9 Angiogenesis

Inhibition of angiogenesis was assessed using Millipore's Millicell μ‐Angiogenesis Inhibition Assay according to the manufacturer's recommendations. CMEC suspensions were seeded in the presence/absence of full‐length Aβ peptides or the N‐ and C‐terminal truncated fragments, in a Millicell μ‐Angiogenesis Slide containing ECMatrix Gel Solution. Sulforaphane was used as a positive control of angiogenesis inhibition. In all cases, tube formation was monitored after 24 h by fluorescent staining with 50 μM Calcein AM and visualized with an EVOS M5000 imaging system. Capillary branches meeting length criteria were counted from 4 randomized images for each treatment.

### 4.10 In vitro blood‐brain barrier permeability

Cerebrovascular endothelial barrier formation was assessed using the ECIS Zθ system (Applied Biophysics). All experimental procedures were performed in 8‐well ECIS (8WE10+, Applied Biophysics) gold plated arrays pre‐treated according to the manufacturer's instructions. A monodisperse solution of CMECs was seeded and monitored for 48 h until the electrical resistance reached a plateau at a frequency of 4000 Hz, indicative of barrier formation. At this point, the cell monolayers were treated with 10 µM solutions of the different Aβ peptides or pre‐aggregated Aβ42 in EBM‐2 media containing 1% FBS and followed for 72 h post‐treatment. Barrier permeability was assessed as a decrease in barrier resistance at 4000 Hz compared with untreated cells.

### 4.11 Statistical analysis

In all cases, graphs are representative of at least 3 independent experiments/group and data are represented as means ±SEM. Normality was evaluated using the Shapiro–Wilk test and statistical significance assessed by one‐way ANOVA followed by Tukey post hoc test using Prism Graph Pad. Differences were considered statistically significant at *p* values ≤0.05.

## CONFLICT OF INTEREST

None.

## AUTHORS CONTRIBUTIONS

SF and JG designed the study. SF and RPR performed experiments and wrote the manuscript. EC and AR performed the structural characterization of the peptides. JG and AR edited and revised the manuscript.

## Data Availability

The data that support the findings of this study are available from the corresponding author, upon reasonable request.
